# Supplementary material for: The Fergana Valley Is an Isolate of Biodiversity: A Discussion of the Endemic Herpetofauna and Description of Two New Species of Alsophylax (Sauria: Gekkonidae) from Eastern Uzbekistan
Source: Animals (Basel). 2023 Aug 4;13(15):2516. doi: 10.3390/ani13152516 (PMC10417568; doi:10.3390/ani13152516)
Supplement: Supplementary file 1 [file animals-13-02516-s001.zip › animals-2479180-supplementary.pdf]

Supplementary Materials S1. Distribution maps of the rare and endemic reptile species in the Fergana Valley. They also show the comparison of historical distribution obtained from literature and specimen data (yellow circles) with the current distribution of these species (red circles) obtained during our field observations.

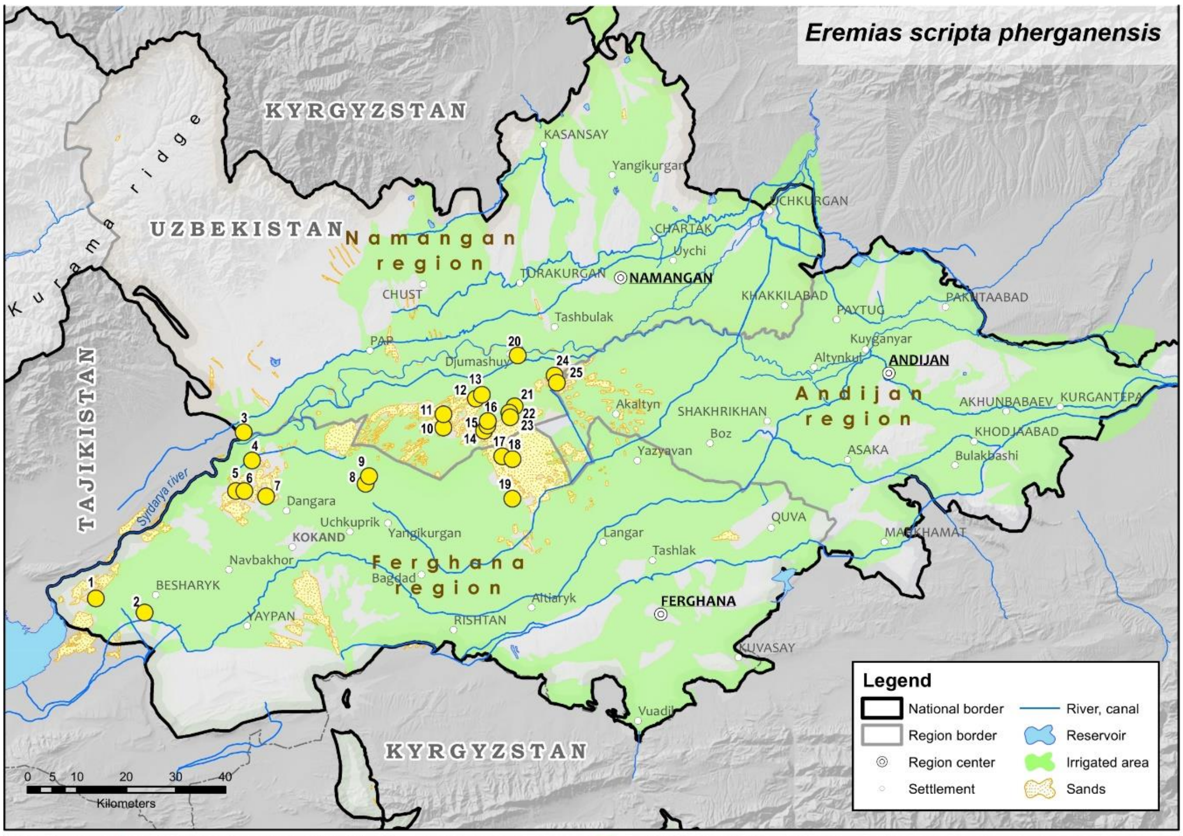

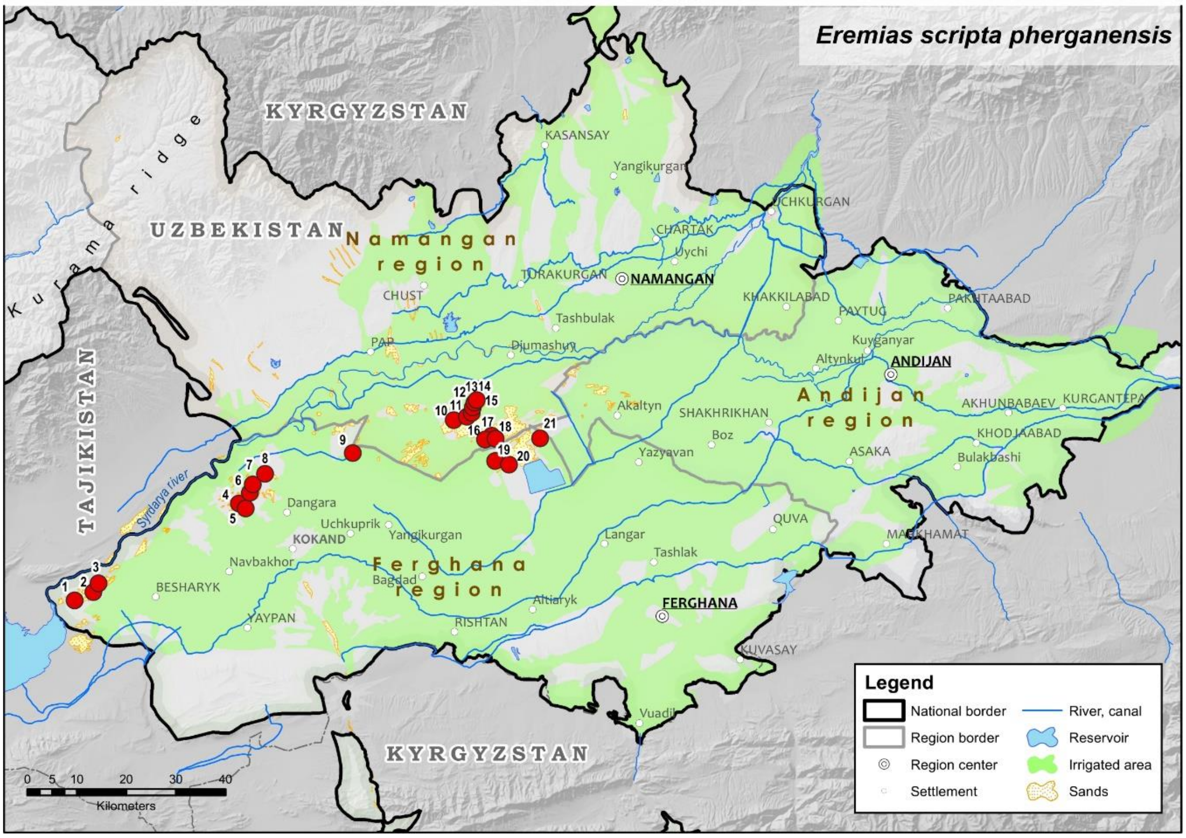

Figure S1. Distribution of *Eremias scripta pherganensis*. Top - Historical distribution of *E. scripta pherganensis*. 1 - Fergana region, 1 km northwest of the village of Kiyali; N 40.439757, E 70.425111 (Bondarenko, 2020), 2 - Fergana region, the vicinity of the Yakkatut village; N 40.403116, E 70.533359 (Nazarov et al., 2016), 3 - Namangan region, the vicinity of the Pungan village; N 40.737059, E 70.799348 (Zakhidov et al., 1970; Vashetko, 1972), 4 - Fergana region, the vicinity of the Kichik Turk village; N 40.685173, E 70.822952 (Nazarov et al., 2016), 5 – Fergana region, 1.5 km south of the Doimabad village; N 40.626043, E 70.812218 (Bondarenko, 2020), 6 - Fergana region, 300 m north of the Doimabad village; N 40.653601, E 70.811835 (Bondarenko, 2020), 7 - Namangan region, the vicinity of the Chinabad village; N 40.636751, E 70.863016 (Nazarov et al., 2016), 8 – Ferghana region, Buvayda railway station; N 40.652524, E 71.102674 (Bogdanov, 1960; Vashetko, 1972), 9 – Ferghana region, Buvayda railway station; N 40.652524, E 71.102674 (Vashetko, Kamalova, 1978), 10 - Fergana region, Yazyavan Nature Sanctuary; N 40.732240, E 71.283636 (Chikin, 2001), 11 – Fergana region, northwestern corner of the Akkum sands; N 40.768983, E 71.285064 (Vashetko E.V.), 12 – Fergana region, Yazyavan Nature Sanctuary; N 40.788981, E 71.373719 (Chikin, 2001), 13 – Fergana region, northern corner of the Akkum sands; N 40.795239, E 71.380336 (Nazarov et al., 2016), 14 – Fergana region, third Takalyk, Akkum sands; N 40.728657, E 71.382980 (Bogdanov, 1960), 15 – Ferghana region, 9 km southwest of Yangiturmysh village; N 40.73305, E 71.38878 (Bondarenko, 2020), 16 - Fergana region, 7.5 km southwest of the Yangiturmysh village; N 40.746837, E 71.38878 (Bondarenko, 2020), 17 – Fergana region, 4.5 km northwest of Uzimchilik village; N 40.685105, E 71.422722 (Bondarenko, 2020), 18 – Ferghana region, Yazyavan Nature Sanctuary; N 40.681588, E 71.453191 (Chikin, 2001), 19 - Fergana region, 40 km along the Kokand-Andijan highway; N 40.597673, E 71.440932 (Nazarov et al., 2016), 20 – Namangan region, vicinity of Dzhumashui village; N 40.865249, E 71.443507 (Nazarov et al., 2016), 21 – Fergana region, Akkum sands; N 40.765894, E 71.450804 (Vashetko and Kamalova, 1978), 22 – Fergana region, 6 km west of Akkum village; N 40.7601, E 71.448175 (Bondarenko, 2020), 23 – Ferghana region, 50 km northeast of Kokand, Akkum sands; N 40.742976, E 71.42823 (Nazarov et al., 2016), 24 – Andijan region, Gurtepa village; N 40.827266, E 71.558435 (Zakhidov et al., 1970; Vashetko, 1974), 25 – Andijan region, Gurtepa village; N 40.827266, E 71.558435 (Vashetko, 1972).

Bottom - Current distribution of *E. scripta pherganensis*. 1 - Fergana region, Kairakum sand massif; N 40.43231, E 70.40362, 2 – Fergana region, Kairakum sand massif; N 40.44350, E 70.41715, 3 – Fergana region, Kairakum sand massif, vicinity of the Shada Kazik village; N 40.468561, E 70.437523, 4 - Fergana region isolated sands in vicinity of the Katta Turk village; N 40.60204, E 70.79998, 5 - Ferghana region, isolated sands in vicinity of the Katta Turk village; N 40.59728, E 70.80398, 6 - Fergana region, isolated sands in vicinity of the Katta Turk village; N 40.63014, E 70.81468, 7 - Fergana region, isolated sands in vicinity of the Katta Turk village; N 40.65065, E 70.81622, 8 - Fergana region, isolated sands in vicinity of the Katta Turk village; N 40.661269, E 70.846181, 9 - Namangan region, isolated sand area in the Mingbulak region; N 40.70778, E 71.07349, 10 – Namangan region, Akkum sands, Mingbulak district; N 40.758935, E 71.316520, 11 – Namangan region, Mingbulak sands; N 40.76508, E 71.34723, 12 – Namangan region, Mingbulak sands; N 40.77025, E

71.35492, 13 – Namangan region, Mingbulak sands; N 40.77548, E 71.35531, 14 - Namangan region, Mingbulak sands; N 40.77555, E 71.35544, 15 - Namangan region, Akkum sands, Mingbulak district; N 40.785461, E 71.355600, 16 - Ferghana region, Yazyavan Nature Sanctuary; N 40.728657, E 71.382980, 17 – Fergana region, Akkum sands; N 40.728657, E 71.382980, 18 – Fergana region, Akkum sands, Yazyavan Nature Sanctuary; N 40.72195, E 71.39295, 19 - Fergana region, Akkum sands, Yazyavan Nature Sanctuary; N 40.68172, E 71.39686, 20 - Fergana region, fish farms in vicinity of Yazyavan Nature Sanctuary; N 40.672887, E 71.432144, 21 – Fergana region, Yazyavan Nature Sanctuary; N 40.712533, E 71.521029.

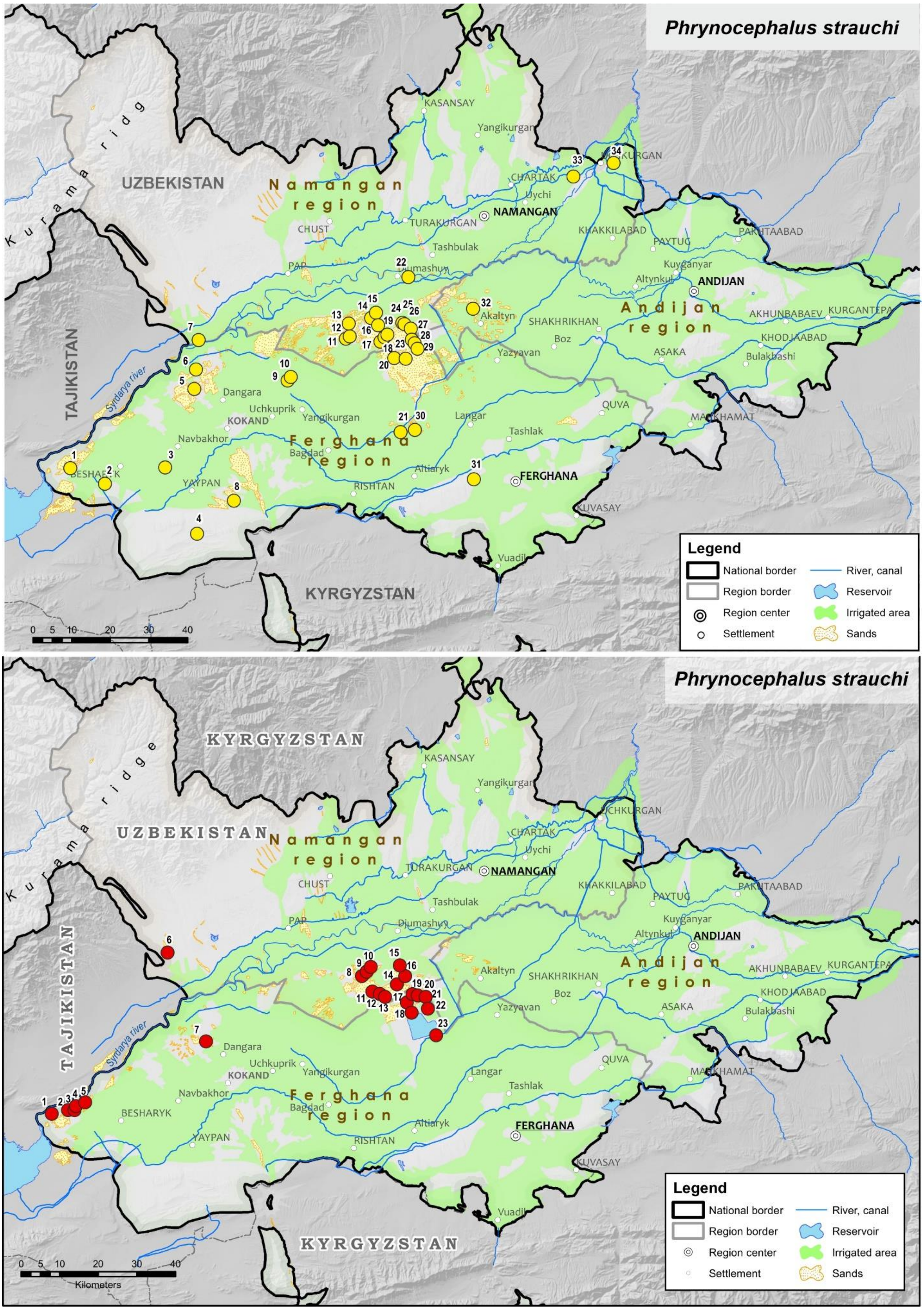

Figure S2. Distribution of *Phrynocephalus strauchi*. Top - Historical distribution of *P. strauchi***.** 1 - Fergana region, 1 km northwest of the Kiyali village; N 40.439757, E 70.425111

(Bondarenko, 2020), 2 – Fergana region, Yakkatut village; N 40.403116, E 70.533359 (Nazarov et al., 2016), 3 – Fergana region, Karasu; N 40.426767, E 70.695001 (Nikol'skii, 1899, 1905), 4 – Fergana region, vicinity of the Shorsu reservoir; N 40.286473, E 70.803834 (Zakhidov et al., 1970), 5 – Fergana region, environs of Chinabad village; N 40.635168, E 70.810849 (Nazarov et al., 2016), 6 – Fergana region, vicinity of Taptik Saray village; N 40.663756, E 70.785014 (Nazarov et al., 2016), 7 – Namangan region, vicinity of Pungan village; N 40.738159, E 70.806581 (Zakhidov et al., 1970), 8 – Fergana region, sands in the vicinity of Shorsu settlement; N 40.359275, E 70.921761 (Vashetko and Kamalova, 1978), 9 – Fergana region, vicinity of Buvayda railway station; N 40.652524, E 71.102674 (Bogdanov, 1960), 10 – Fergana region, vicinity of Buvayda railway station; N 40.652524, E 71.102674 (Vashetko, Kamalova, 1978), 11 – Fergana region, Yazyavan Nature Sanctuary; N 40.732240, E 71.283636 (Chikin, 2001), 12 – Ferghana region, Yazyavan Nature Sanctuary; N 40.732240, E 71.283636 (Nazarov et al., 2016), 13 – Fergana region, northwestern corner of the Akkum sands; N 40.768983, E 71.285064 (Nazarov et al., 2016), 14 – Namangan region, Akkum sands, Mingbulak district; N 40.785461, E 71.355600 (Zakhidov et al., 1970), 15 – Fergana region, Akkum sands; N 40.790823, E 71.372403 (Zakhidov et al., 1970), 16 – Fergana region, between Kokand and Namangan cities; N 40.774402, E 71.378652 (Bogdanov, 1960), 17 – Fergana region, 9 km southwest of Yangiturmysh village; N 40.73305, E 71.38878 (Bondarenko, 2020), 18 – Fergana region, Third Takalyk, Akkum sands; N 40.728657, E 71.382980 (Collection of the Institute of Zoology of the Academy of Sciences of Uzbekistan; Bogdanov, 1960), 19 - Fergana region, 7.5 km southwest of the village of Yangiturmysh; N 40.746837, E 71.38878 (Bondarenko, 2020), 20 – Fergana region, 4.5 km northwest of Uzimchilik village; N 40.685105, E 71.422722 (Bondarenko, 2020), 21 – Fergana region, Kiziltepe village; N 40.51293, E 71.437156 (Nazarov et al., 2016), 22 – Namangan region, vicinity of Dzhumashui village; N 40.865249, E 71.443507 (Nazarov et al., 2016), 23 – Fergana region, Yazyavan Nature Sanctuary; N 40.681588, E 71.453191 (Zakhidov et al., 1970), 24 – Fergana region, Akkum sands; N 40.765894, E 71.450804 (Oral communication by Yusupov V.), 25 – Fergana region, Akkum sands; N 40.765894, E 71.450804 (Vashetko and Kamalova, 1978), 26 – Fergana region, Akkum sands; N 40.765894, E 71.450804 (Zakhidov et al., 1970), 27 – Fergana region, Akkum sands; N 40.765894, E 71.450804 (Vashetko E.V., Kamalova Z.Ya.), 28 – Fergana region, 6 km west of Akkum village; N 40.7601, E 71.448175 (Bondarenko, 2020), 29 – Fergana region, 50 km northeast of Kokand, Akkum sands; N 40.733733, E 71.477332 (Nazarov et al., 2016), 30 – Fergana region, sands west of Fergana; N 40.513936, E 71.481639 (Bogdanov, 1960; Vtorov and Pereshkolnik, 1970), 31 – Fergana region, 8 km west of the Fergana city; N 40.397032, E 71.647722 (Zakhidov et al., 1970), 32 – Fergana region, sands between Kokand and Andijan cities, Akkum sands; N 40.799133, E 71.672441 (Nazarov et al., 2016), 33 – Namangan region, vicinity of Terekurgan village; N 41.099217, E 71.989242 (Bogdanov, 1960), 34 – Namangan region, vicinity of Uchkurgan village; No. 41.116937, E 72.110092 (Yakovleva, 1964).

Bottom Current distribution of *P. strauchi*. 1 – Fergana region, variegated outcrops near the Kairakum sands on the border with Tajikistan, near the Syrdarya River; N 40.467504, E 70.378391, 2 – Fergana region, Kairakum sand massif, vicinity of the Shada Kazik village;

N 40.465447, E 70.428428, 3 – Fergana region, Kairakum sand massif, vicinity of the Shada Kazik village; N 40.468561, E 70.437523, 4 – Fergana region, Kairakum sand massif, vicinity of the Shada Kazik village; N 40.470722, E 70.458255, 5 – Fergana region, Kairakum sand massif, vicinity of the Shada Kazik village; N 40.476506, E 70.473750, 6 – Uzbekistan, Namangan region, Pap foothills; N 40.831743, E 70.736846, 7 - Fergana region, Fergana region, Dangara district, neighborhood of the villages of Karasaray and Daynobod; N 40.627351, E 70.870932, 8 – Namangan region, Akkum sands, Mingbulak region; N 40.76508, E 71.34723, 9 – Namangan region, Akkum sands, Mingbulak district; N 40.77548, E 71.35531, 10 – Namangan region, Akkum sands, Mingbulak district; N 40.785461, E 71.355600, 11 – Fergana region, Akkum sands; N 40.728657, E 71.382980, 12 – Fergana region, Akkum sands, Yazyavan Nature Sanctuary; N 40.72195, E 71.39295, 13 - Fergana region, fish farms near Yazyavan Nature Sanctuary; N 40.708329, E 71.405250, 14 – Fergana region, Akkum sands; N 40.741991, E 71.444597, 15 – Namangan region, Akkum sands, Mingbulak district; N 40.796442, E 71.457752, 16 - Namangan region, foothills of the Kurama ridge; N 40.7673, E 71.4674, 17 - Fergana region, Akkum sands, Yazyavan Nature Sanctuary; N 40.67628, E 71.48158, 18 - Ferghana region, Akkum sands, Yazyavan Nature Sanctuary; N 40.67595, E 71.48220, 19 – Fergana region, Yazyavan Nature Sanctuary; N 40.713603, E 71.471392, 20 – Fergana region, Yazyavan Nature Sanctuary; N 40.716225, E 71.487536, 21 – Fergana region, Yazyavan Nature Sanctuary; N 40.712533, E 71.521029, 22 - Fergana region, fish farms near Yazyavan Nature Sanctuary; N 40.684699, E 71.532504, 23 – Ferghana region, on the bank of the Yazyavan reservoir.; N 40.624253, E 71.551309.

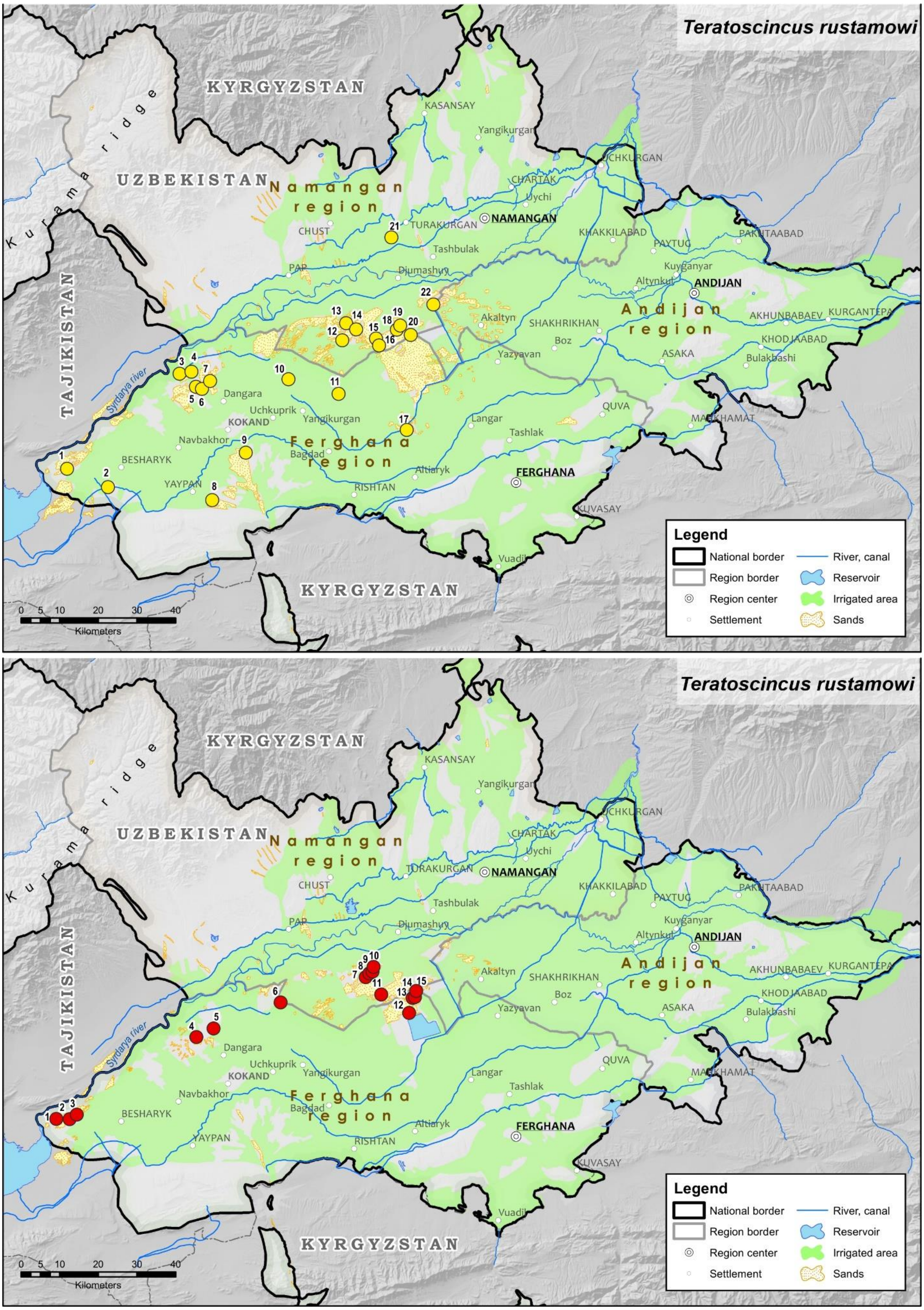

Figure S3. Distribution of *Teratoscincua rustamowi*. Top - Historical distribution of *T. rustamowi*. 1 – Fergana region, 1 km northwest of the Kiyali village; N 40.439757, E

70.425111 (Bondarenko, 2020), 2 – Fergana region, Yakkatut village; N 40.403116, E 70.533359 (Nazarov et al., 2016; Nazarov et all, 2017), 3 – Fergana region, vicinity of Taptik Saray village; N 40.663756, E 70.785014 (Nazarov et al., 2016; Nazarov et all, 2017), 4 – Fergana region, 300 m north of Doimabad settlement; N 40.653601, E 70.811835 (Bondarenko, 2020), 5 - Fergana region, 1.5 km south of the Doimabad village; N 40.626043, E 70.812218 (Bondarenko, 2020), 6 - Fergana region, 30 km west of the city of Kokand, vicinity of Chinabad village; N 40.622466, E 70.832736 (Nazarov et al., 2016; Nazarov et all, 2017), 7 – Fergana region, environs of Chinabad village; N 40.636751, E 70.863016 (Nazarov et al., 2016; Nazarov et all, 2017), 8 – Fergana region, vicinity of Yaipan village; N 40.366406, E 70.861747 (Nazarov et al., 2016; Nazarov et all, 2017), 9 – Fergana region, vicinity of Kokand city; N 40.473962, E 70.956332 (Chernov, 1949; Nazarov et al., 2016; Nazarov et all, 2017), 10 – Ferghana region, vicinity of Buvayda railway station; N 40.652524, E 71.102674 (Bogdanov, 1960), 11 – Fergana region, vicinity of Aktepe; N 40.605069, E 71.230476 (Nazarov et al., 2016; Nazarov et all, 2017), 12 – Ferghana region, Yazyavan Nature Sanctuary; N 40.732240, E 71.283636 (Chikin, 2001), 13 – Fergana region, Northwestern corner of the Akkum sands; N 40.768983, E 71.285064 (Nazarov et al., 2016; Nazarov et all, 2017), 14 – Fergana region, between the Kokand and Dzhumashui cities; N 40.743810, E 71.310389 (Szczerbak and Golubev, 1986), 15 – Fergana region, 9 km southwest of Yangiturmysh village; N 40.73305, E 71.38878 (Bondarenko, 2020), 16 – Fergana region, Third Takalyk, Akkum sands; N 40.728657, E 71.382980 (Bogdanov, 1960; Nazarov et al., 2016; Nazarov et all, 2017), 17 – Fergana region, Kiziltepe village; N 40.51293, E 71.437156 (Zakhidov et al., 1970), 18 – Fergana region, Akkum sands; N 40.765894, E 71.450804 (Vashetko and Kamalova, 1978), 19 – Fergana region, Akkum sands; N 40.765894, E 71.450804 (Nazarov et al., 2016; Nazarov et all, 2017), 20 – Fergana region, 6 km west of Akkum village; N 40.7601, E 71.448175 (Bondarenko, 2020), 21 – Namangan region, sands in the vicinity of Shahidon village; N 40.963935, E 71.425912 (Zakhidov et al., 1970), 22 – Andijan region, Gurtepa settlement; N 40.827266, E 71.558435 (Zakhidov et al., 1970).

Current distribution of *T. rustamowi.* 1 – Fergana region, variegated outcrops near the Kairakum sands on the border with Tajikistan, near the Syrdarya river; N 40.45729, E 70.38650, 2 – Fergana region, Kairakum sand massif; N 40.43754, E 70.40433, 3 – Fergana region, Kairakum sand massif; N 40.44350, E 70.41715, 4 - Fergana region, isolated sands near the Katta Turk village; N 40.63035, E 70.81481, 5 – Fergana region, vicinity of Dangara settlement; N 40.651748, E 70.875879, 6 - Namangan region, isolated sandy area in the Mingbulak region; N 40.70778, E 71.07349, 7 – Namangan region, Mingbulak sands; N 40.77025, E 71.35492, 8 – Namangan region, Akkum sands, Mingbulak district; N 40.77548, E 71.35531, 9 - Namangan region, sandy area in the Mingbulak region; N 40.77555, E 71.35544, 10 - Namangan region, Akkum sands, Mingbulak region; N 40.785461, E 71.355600, 11 – Fergana region, Akkum sands; N 40.728657, E 71.382980, 12 – Fergana region, Akkum sands, Yazyavan Nature Sanctuary; N 40.67595, E 71.48220, 13 – Fergana region, Akkum sands, Yazyavan Nature Sanctuary; N 40.71559, E 71.48831, 14 – Fergana region, Akkum sands, riparian forest; N 40.71577, E 71.48851, 15 – Fergana region, Yazyavan Nature Sanctuary; N 40.716225, E 71.487536.

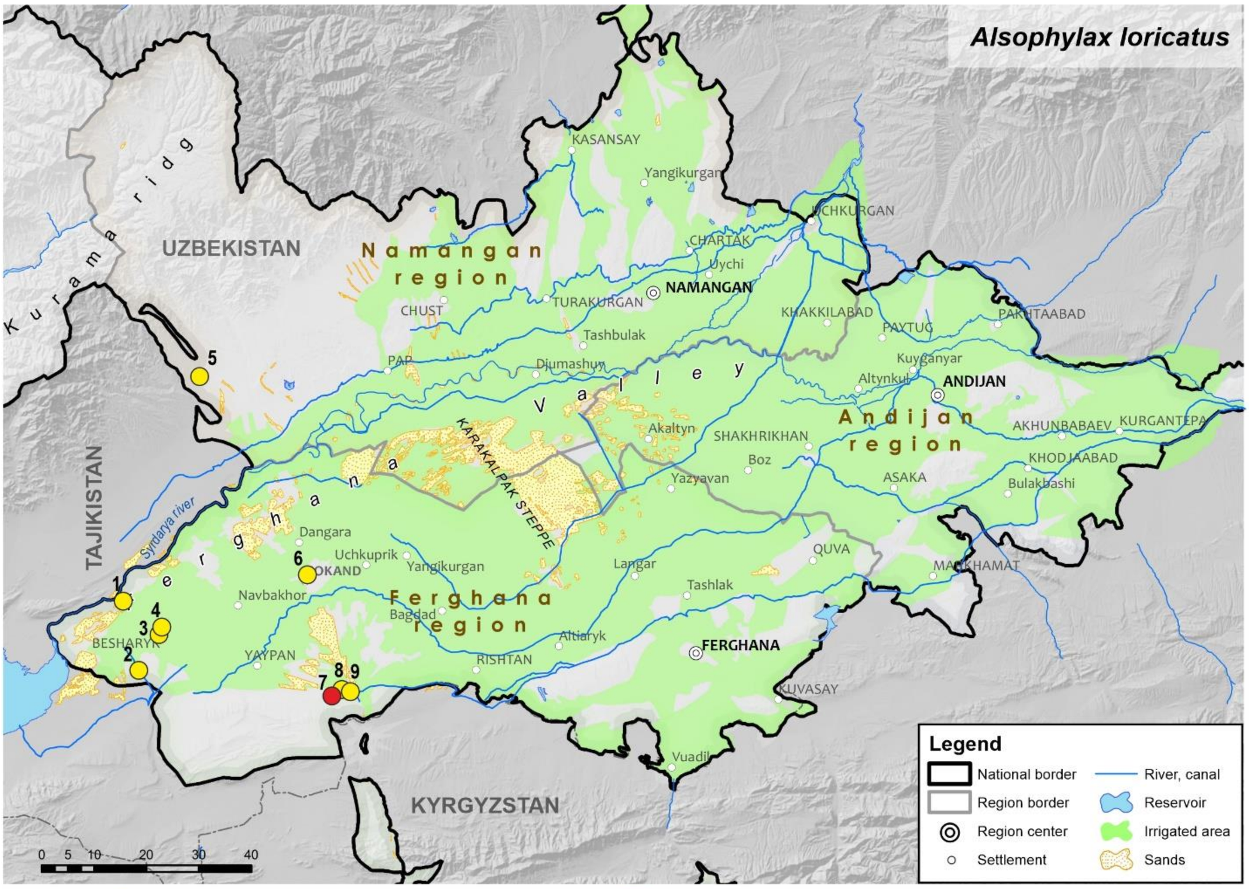

Alsophylax loricatus
UZBEKISTAN
TAJIKISTAN
KYRGYZSTAN
Kurama ridg
Namangan region
Ferghana region
Andijan region
Ferghana Valley
KARAKALPAK STEPPE
Syrdarya river
NAMANGAN
ANDIJAN
FERGHANA
KOKAND
Legend
National border
Region border
Region center
Settlement
River, canal
Reservoir
Irrigated area
Sands
0 5 10 20 30 40

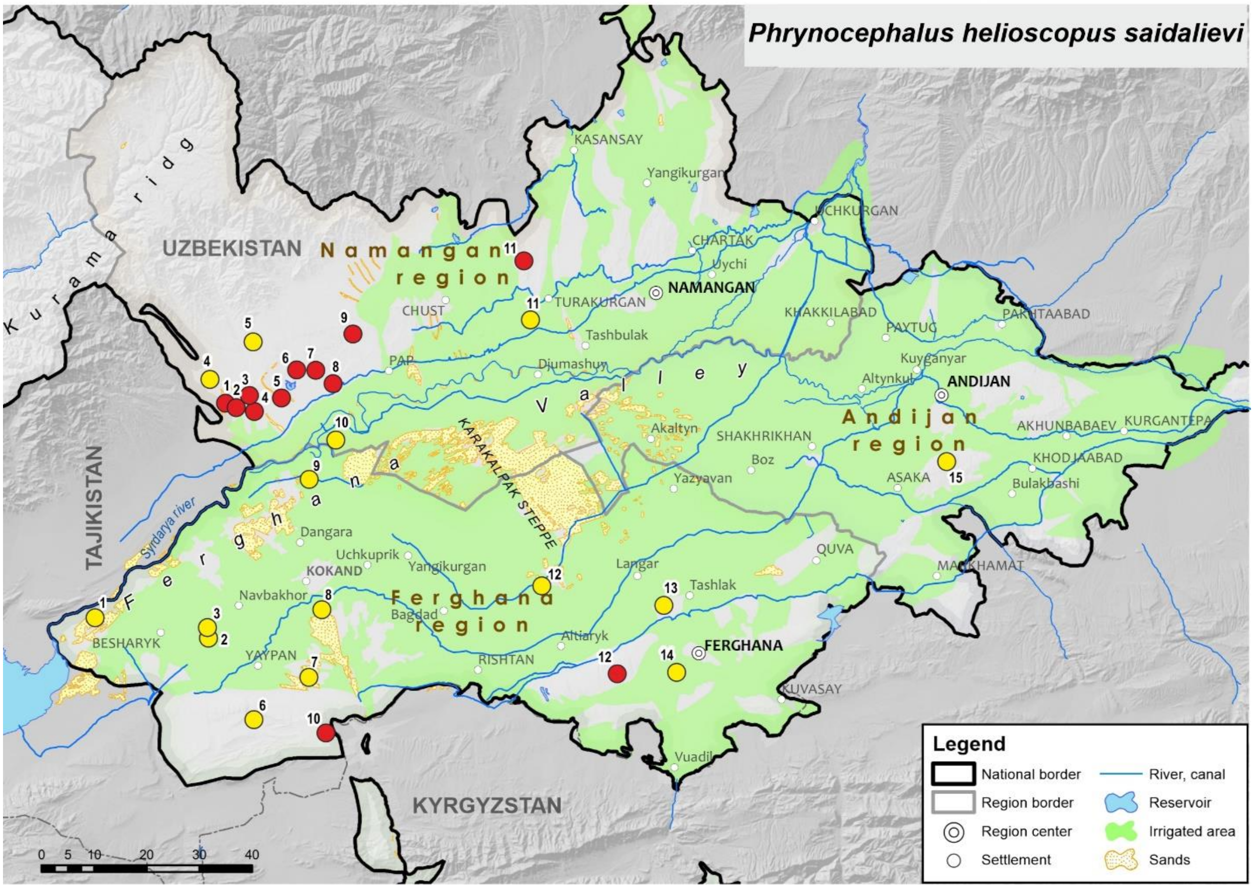

Phrynocephalus helioscopus saidalievi
UZBEKISTAN
TAJIKISTAN
KYRGYZSTAN
Kurama ridg
Namangan region
Ferghana region
Andijan region
Ferghana Valley
KARAKALPAK STEPPE
Syrdarya river
NAMANGAN
ANDIJAN
FERGHANA
KOKAND
Legend
National border
Region border
Region center
Settlement
River, canal
Reservoir
Irrigated area
Sands
0 5 10 20 30 40

Figure S4. Top - Distribution of *Alsophylax loricatus*. Historical distribution (yellow circles)

1 – Fergana region, Naukat village; N 40.494395, E 70.532071 (Nazarov et al., 2016), 2 – Fergana region, Kirov forestry; N 40.375169, E 70.564515 (Szczerbak and Golubev, 1986), 3 – Fergana region, Besharyk settlement; N 40.436147, E 70.611379 (Collection of the Institute of Zoology of the Academy of Sciences of Uzbekistan, Kamalova Z.Ya.), 4 – Fergana region, Kurgancha village; N 40.448689, E 70.618074 (Nazarov et al., 2016), 5 – Namangan region, watershed of the Angren and Syrdarya rivers; N 40.878318, E 70.713979 (Bogdanov, 1960), 6 – Fergana region, Kokand city; N 40.533416, E 70.943715 (Nazarov et al., 2016), 7 – Fergana region, fortress in the Sary-Kurgan village; N 40.334791, E 71.021476. Current distribution (red circles) 7 – Fergana region, a fortress in the Sary-Kurgan village; N 40.334791, E 71.021476, N 40.334791, E 71.021476 (Nazarov et al., 2016).

Bottom – Distribution of *Phrynocephalus helioscopus saidalievi*. Historical distribution (yellow circles). 1 – Fergana region, Kairakum sands; N 40.467041, E 70.450876 (Nazarov et al., 2016), 2 – Fergana region, Karasu; N 40.426767, E 70.695001 (Nazarov et al., 2016), 3 – Fergana region, Namuna village; N 40.445713, E 70.705816 (Collection of the Institute of Zoology of the Academy of Sciences of Uzbekistan, Kamalova Z.Ya.), 4 – Namangan region, watershed of the Angren and Syrdarya rivers; N 40.878318, E 70.713979 (Bogdanov, 1960), 5 – Namangan region, Pap foothills, environs of the villages of Chodana and Koshminora; N 40.942478, E 70.788705 (Nazarov et al., 2016), 6 – Fergana region, vicinity of the Shorsu reservoir; N 40.286473, E 70.803834 (Collection of the Institute of Zoology of the Academy of Sciences of Uzbekistan, Kamalova Z.Ya.), 7 – Fergana region, sands in the vicinity of Shorsu settlement; N 40.359275, E 70.921761 (Collection of the Institute of Zoology of the Academy of Sciences of Uzbekistan, Vashetko E.V., Kamalova Z.Ya.), 8 – Fergana region, vicinity of the Kokand city; N 40.473962, E 70.956332 (Nazarov et al., 2016), 9 – Fergana region, Urganchi village; N 40.695391, E 70.936677 (Nazarov et al., 2016), 10 – Namangan region, 25 km north of the Kokand city; N 40.768914, E 70.987932 (Nazarov et al., 2016), 11 – Namangan region, sands in the vicinity of Shahidon village; N 40.963935, E 71.425912 (Collection of the Institute of Zoology of the Academy of Sciences of Uzbekistan, Vashetko E.V., Kamalova Z.Ya.), 12 – Fergana region, Between Margelan and Kokand cities; N 40.525252, E 71.436159 (Bogdanov, 1960), 13 – Fergana region, Margelan city; N 40.469905, E 71.71631 (Shchelkanovtsev, 1897), 14 – Fergana region, environs of the Fergana city; N 40.354465, E 71.752864 (Nazarov et al., 2016), 15 – Andijan region, in the vicinity of the Andijan city; N 40.693625, E 72.365534 (Bogdanov, 1960).

Current distribution (red circle). 1 – Namangan region, Pap foothills; N 40.831427, E 70.741621, 2 – Namangan region, Pap foothills; N 40.834267, E 70.747901, 3 - Namangan region, foothills of the Kurama ridge; N 40.84706, E 70.781136, 4 - Namangan region, foothills of the Kurama ridge; N 40.820735, E 70.792477, 5 – Namangan region, Pap foothills, Khanabad military training ground; N 40.84456, E 70.85038, 6 – Fergana region, Pap foothills; N 40.891349, E 70.884367, 7 – Namangan region, Pap foothills; N 40.8906, E 70.92979, 8 - Namangan region, Pap foothills, road to the Mazar village; N 40.86665, E 70.97068, 9 – Namangan region, Pap foothills, vicinity of Uygursay village; N 40.95126, E

71.01833, 10 – Fergana region, vicinity of the Shorsay reservoir; N 40.2645, E 70.938062, 11 – Namangan region, Pap foothills; N 41.067757, E 71.412176, 12 – Fergana region, vicinity of the Altyaryk village; N 40.355251, E 71.591541.
